# Hypothalamus-pituitary-adrenal and gut-brain axes in biological interaction pathway of the depression

**DOI:** 10.3389/fnins.2025.1541075

**Published:** 2025-02-06

**Authors:** Amanda Gollo Bertollo, Camila Ferreira Santos, Margarete Dulce Bagatini, Zuleide Maria Ignácio

**Affiliations:** Laboratory of Physiology Pharmacology and Psychopathology, Graduate Program in Biomedical Sciences, Federal University of Fronteira Sul, Chapecó, Brazil

**Keywords:** hypothalamus-pituitary-adrenal axis, gut-brain axis, gut microbiota, depression, stress response

## Abstract

The hypothalamus-pituitary-adrenal (HPA) and gut-brain axes are vital biological pathways in depression. The HPA axis regulates the body's stress response, and chronic stress can lead to overactivation of the HPA axis, resulting in elevated cortisol levels that contribute to neuronal damage, particularly in regions such as the hippocampus and prefrontal cortex, both of which are involved in mood regulation and mental disorders. In parallel, the gut-brain axis, a bidirectional communication network between the gut microbiota and the central nervous system, influences emotional and cognitive functions. Imbalances in gut microbiota can affect the HPA axis, promoting inflammation and increasing gut permeability. This allows endotoxins to enter the bloodstream, contributing to neuroinflammation and altering neurotransmitter production, including serotonin. Since the majority of serotonin is produced in the gut, disruptions in this pathway may be linked to depressive symptoms. This review explores the interplay between the HPA axis and the gut-brain axis in the context of depression.

## Graphical Abstract

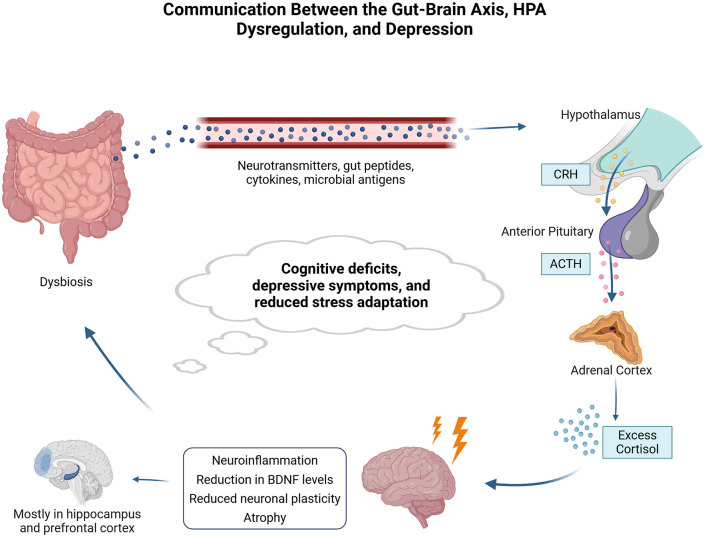

## Introduction

Major Depressive Disorder (MDD) is a complex psychiatric condition that affects millions of people worldwide, with a significant impact on social and occupational functioning (Kessler and Bromet, 2013). It is characterized by a profound sense of sadness, a marked loss of interest or pleasure in previously moderate activities, and a range of cognitive and physical symptoms, such as fatigue, feelings of worthlessness, difficulty concentrating, and changes in appetite and sleep (American Psychiatric Association, 2022). These symptoms lead to severe impairments in quality of life, making MDD a leading cause of disability globally (World Health Organization, 2023).

Recent advances have expanded our understanding of MDD beyond the traditional focus on neurotransmitter imbalances, particularly dysregulation of serotonin and dopamine. The modern view includes a broader analysis of the gut-brain axis (GBA) and the hypothalamic-pituitary-adrenal (HPA) axis, as well as the mechanisms underlying the inflammatory response that may contribute to the development and persistence of depressive symptoms (Otte et al., 2016).

The HPA axis plays a crucial role in the body's stress response by regulating cortisol release. Chronic dysregulation of the HPA axis, often seen in individuals with MDD, results in prolonged exposure to cortisol, leading to atrophy of the hippocampus, a brain region involved in mood regulation (Pariante and Lightman, 2008). This neuroendocrine pathway has been shown to interact with inflammatory processes and oxidative stress, further exacerbating the depressive symptoms and cognitive impairments associated with MDD (Zunszain et al., 2013).

In patients with MDD, hyperactivity of the HPA axis is frequently observed, resulting in elevated and persistent cortisol levels. While cortisol is necessary for an adaptive stress response, its chronic and excessive production can have neurotoxic effects, including reduced hippocampal volume, a brain area crucial for emotional regulation and memory. The hippocampus is a central brain structure for emotional regulation and memory, and its atrophy is associated with impairing these functions in people with depression (Videbech and Ravnkilde, 2004; Knezevic et al., 2023).

Furthermore, under normal conditions, high cortisol levels should activate harmful feedback mechanisms to inhibit further production of this hormone. However, in individuals with MDD, there is evidence that this negative feedback is altered, resulting in sustained activation of the HPA axis and perpetuation of the hypercortisolism state (Holsen et al., 2011). This prolonged state of HPA axis activation contributes to a series of physiological consequences, including amplifying the inflammatory response, which, as previously mentioned, affects neuroplasticity and exacerbates depressive symptoms (Kinlein et al., 2015).

The GBA is a bidirectional communication pathway between the gastrointestinal tract and the central nervous system (CNS). This axis involves a complex network of neurochemical, immunological, and hormonal interactions that allow the gut and brain to communicate continuously and dynamically. Gut dysbiosis, an imbalance in the microbial composition in the gut (Sorboni et al., 2022), has been linked to altered neurotransmitter production and increased inflammation, which have been implicated in depressive symptoms (Kelly et al., 2016). Furthermore, changes in the gut microbiota can activate the immune system, increasing pro-inflammatory cytokines that may disrupt normal brain function and contribute to MDD (Cryan and Dinan, 2012).

The gut microbiome plays a fundamental role in GBA interaction, influencing digestive health, brain function, and human behavior. Recent studies indicate that the gut microbiome, composed of trillions of microorganisms, not only participates in the digestion of food but also synthesizes neurotransmitters such as serotonin, dopamine, and GABA (gamma-aminobutyric acid), which are crucial for regulating mood and anxiety, two aspects closely related to MDD (Dinan and Cryan, 2017). For example, about 90% of the body's serotonin is produced in the gut, highlighting the importance of this pathway for mental health (Yano et al., 2015).

Intestinal dysbiosis, an imbalance in the composition of gut bacteria, has been consistently associated with the development of mood disorders, including MDD (Sampson and Mazmanian, 2015). This imbalance can increase intestinal permeability, allowing lipopolysaccharides (LPS), components of bacterial cell walls, to enter the systemic circulation. This triggers a chronic inflammatory response that can have deleterious effects on the CNS. Pro-inflammatory cytokines such as interleukin (IL)-6 and tumor necrosis factor-alpha (TNF-α) can cross the blood-brain barrier, negatively influencing neuroplasticity and neuronal function, thereby contributing to depressive symptomatology (Kouba et al., 2024; Liu F. et al., 2024).

The GBA and HPA axes do not function in isolation. On the contrary, they are interconnected, and their interaction is central to understanding MDD. Chronic inflammation, promoted by intestinal dysbiosis and prolonged HPA axis activation, plays a central role in the pathophysiology of MDD. This systemic inflammation can promote neuroinflammation, negatively affecting the neural networks involved in mood regulation, memory, and cognition (Miller and Raison, 2016). Inflammation can also compromise the integrity of the blood-brain barrier, allowing inflammatory molecules, such as cytokines, to enter the brain and directly impact neurons and glial cells (Varatharaj and Galea, 2017).

Thus, the complex interaction between the GBA and HPA axes and the underlying mechanisms offers a more holistic view of the MDD pathophysiology. This expanded understanding suggests integrative therapeutic approaches may be more effective, focusing on the CNS and considering gut health and stress regulation. Interventions such as modulation of the gut microbiome through specific diets, probiotics, and prebiotics, as well as strategies to normalize HPA axis function, such as stress reduction techniques and cognitive-behavioral therapy, offer new possibilities for treating and preventing MDD. Thus, a multidisciplinary approach that considers the complex interactions between body and mind may be the key to effectively managing this debilitating condition.

## Major depressive disorder

MDD is a debilitating psychiatric condition that affects a significant portion of the world's population and is considered one of the leading causes of disability and reduced quality of life (Ferrari et al., 2013). In addition to symptoms such as intense sadness, lack of interest in previously pleasurable activities, fatigue, and changes in appetite and sleep, depression can lead to severe cognitive and socioeconomic impairments. The public health impacts of MDD are broad, including increased use of health services, absenteeism, loss of productivity, and an elevated risk of suicide, especially among young adults (Siu et al., 2016).

WHO projections indicate that, by 2030, depression could become the most prevalent disease globally (Liu et al., 2020). In 2015, more than 80% of depression-related deaths occurred in low- and middle-income countries, where MDD accounted for 25.3 and 33.5% of years lost due to disability, respectively (Corea Del Cid, 2021). This scenario underscores the importance of effective and accessible interventions, especially in regions with limited resources.

In addition to directly impacting individuals' health, MDD imposes a substantial economic burden on society. Direct costs include medical treatment and prolonged care, while indirect costs are associated with loss of productivity and absenteeism. The global economic impact of depression is estimated at trillions of dollars per year (Corea Del Cid, 2021), highlighting the urgent need to develop and implement more effective prevention and treatment strategies.

The understanding of MDD's pathophysiological mechanisms has evolved considerably, highlighting a multifactorial etiology involving the interaction of genetic, neurobiological, psychosocial, and environmental factors. Traditionally, depression has been associated with imbalances in brain neurotransmitters such as serotonin, norepinephrine, and dopamine, which are essential for regulating mood, sleep, and appetite. However, the variability in response to antidepressant treatment that modulates these neurotransmitters suggests that other biological pathways also play a crucial role in the development of MDD. Freudian psychoanalysis, for example, conceptualizes depression as a state akin to mourning, characterized by a reduction in self-esteem, disinterest in the external world, loss of the capacity to love, and inhibition of productivity (Corea Del Cid, 2021).

One of the most widely accepted theories today is that MDD is related to alterations in synaptic plasticity, or the brain's ability to strengthen or weaken connections between neurons in response to experiences. Neuroplasticity is fundamental for emotional adaptation and learning. Chronic stress, a known risk factor for depression, can compromise this plasticity by reducing the levels of neurotrophic factors such as brain-derived neurotrophic factor (BDNF). Studies indicate that low BDNF levels are associated with hippocampal atrophy, a brain region crucial for emotional regulation and memory, in people with MDD (Duman et al., 2016).

Another important aspect of MDD pathophysiology involves systemic inflammation and neuroinflammation. Patients with MDD often present elevated levels of inflammatory markers such as C-reactive protein (CRP), IL-6, and TNF-α. Chronic inflammation can affect the brain by crossing the blood-brain barrier and inducing an inflammatory response within the CNS. This neuroinflammation can damage neurons, compromise synaptic plasticity, and alter neurotransmitter function, exacerbating depressive symptoms (Miller and Raison, 2016).

Oxidative stress also plays a crucial role in the pathophysiology of MDD. It results from an imbalance between reactive oxygen species (ROS) production and the body's ability to neutralize them with antioxidants. The excessive accumulation of ROS can damage lipids, proteins, and DNA in brain cells, contributing to neuronal dysfunction and cell death, processes strongly associated with depressive symptoms (Maes et al., 2011).

These advances in understanding the underlying mechanisms of MDD expand scientific knowledge and point to potential therapeutic targets for developing new treatment approaches that may be more effective and tailored to the needs of affected individuals.

## Hypothalamus-pituitary-adrenal axis

The HPA axis plays a central role in the body's stress response and mood regulation, fundamental for understanding the biological mechanisms involved in depression. The HPA axis is activated when the hypothalamus releases corticotropin-releasing hormone (CRH) in response to stress. This hormone stimulates the pituitary gland to secrete adrenocorticotropic hormone (ACTH), which, when circulating through the blood system, stimulates the adrenal glands to release glucocorticoids, such as cortisol, the main stress hormone in humans (Buttenschøn et al., 2017).

A key component of HPA axis regulation is negative feedback, in which cortisol inhibits the production of CRH and ACTH, modulating their levels. Under conditions of chronic stress, this mechanism can break down, generating hyperactivity of the HPA axis, often associated with depression and contributing to symptoms such as apathy, demotivation, and fatigue (Dedovic et al., 2009). Studies suggest that genetic factors may influence susceptibility to HPA axis dysfunction, increasing the predisposition to depression (Buttenschøn et al., 2017).

Prolonged exposure to cortisol can damage structural regions such as the hippocampus, which is critical for memory and learning, and the prefrontal cortex, which is responsible for emotional control and decision-making. Furthermore, the amygdala, which is involved in emotional processing, can become hyperactive, exacerbating symptoms of anxiety and emotional reactivity (Mello et al., 2003; Geerlings and Gerritsen, 2017).

Another critical aspect of HPA axis dysfunction in MDD is the desensitization of glucocorticoid receptors (GR) in target tissues, including the brain. GRs are responsible for mediating the effects of cortisol and play a vital role in the negative feedback of the HPA axis. Desensitization of these receptors can result in an inadequate response to cortisol, further increasing the secretion of this hormone and exacerbating systemic inflammation and neuroinflammation (Pariante, 2004). The dysfunction of the HPA axis can affect several biological mechanisms crucial for mental and physical health. One of the main ones is neurogenesis, the process of forming new neurons in the brain. Elevated cortisol has been associated with suppressing neurogenesis, particularly in the hippocampus. Studies suggest that reduced neurogenesis may contribute to the cognitive and memory deficits often observed in patients with MDD (Lucassen et al., 2010).

Additionally, elevated cortisol can promote inflammation by inducing the production of pro-inflammatory cytokines such as IL-1, IL-6, and TNF-α. These cytokines can cross the blood-brain barrier and contribute to neuroinflammation, which is implicated in the pathogenesis of depression and other neuropsychiatric conditions (Miller and Raison, 2016).

Another significant biological effect of HPA axis dysfunction is the alteration of synaptic plasticity, which is essential for emotional adaptation and learning. Elevated cortisol can interfere with the signaling of BDNF, a protein crucial for the survival and growth of neurons. Reduced levels of BDNF and the consequent decrease in synaptic plasticity have been associated with the severity of depressive symptoms and resistance to antidepressant treatment (Duman and Monteggia, 2006).

The relationship between the HPA axis and the GBA is complex and bidirectional, with significant implications for understanding the pathophysiology of MDD. By activating the HPA axis, chronic stress can lead to alterations in the gut microbiome, promoting dysbiosis and an imbalance in the composition of gut bacteria. Dysbiosis, in turn, can increase intestinal permeability, allowing bacterial components such as LPS to enter the systemic circulation and induce a chronic inflammatory response (Foster and McVey Neufeld, 2013). This systemic inflammation can exacerbate HPA axis dysfunction by increasing the production of pro-inflammatory cytokines, which can cross the blood-brain barrier and directly affect the brain, contributing to neuroinflammation and the perpetuation of depressive symptoms. Moreover, the gut microbiome influences the production of neurotransmitters and other neuroactive substances, such as serotonin, which plays a crucial role in mood regulation and can be modulated by cortisol and inflammatory cytokines (Moreira et al., 2023).

Therefore, HPA axis dysfunction can initiate a negative feedback loop involving both systemic inflammation and alterations in the gut microbiome, exacerbating MDD symptoms and contributing to treatment resistance. Understanding this complex interaction suggests that therapeutic approaches targeting both HPA axis regulation and gut microbiome modulation may be essential to effectively treat MDD.

## Gut-brain axis—GBA

**Figure 1:**
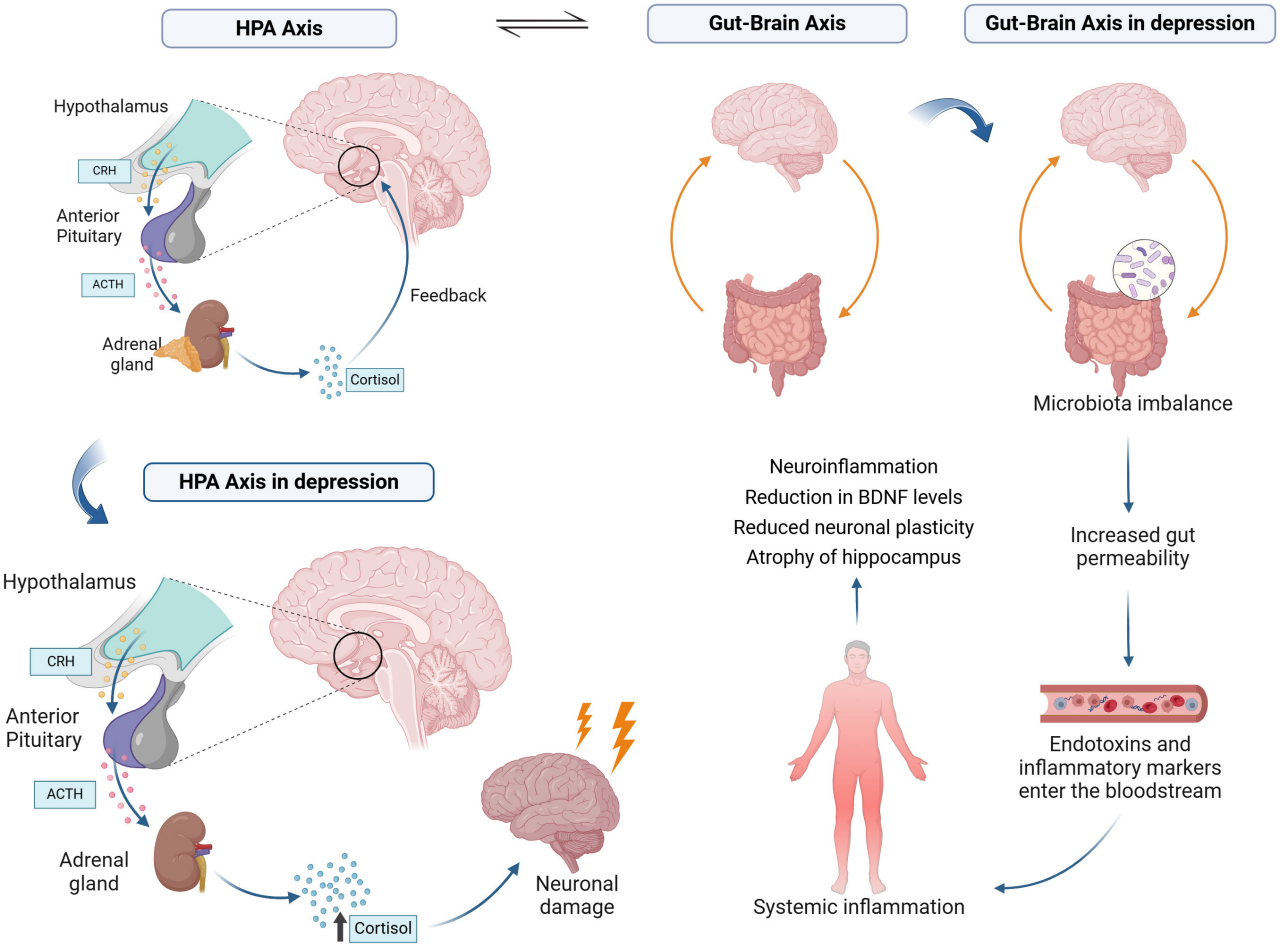
The gut-brain axis (GBA) involves bidirectional communication via neural, immune, and endocrine pathways. The neural pathway operates through the vagus nerve and enteric nervous system, influencing brain function via neurotransmitters like serotonin and GABA. The immune pathway involves cytokine signaling from gut microbiota interactions with immune cells. The endocrine pathway includes hormones like ghrelin, leptin, and SCFAs (short-chain fatty acids) affecting mood and cognition. External factors such as diet, stress, and antibiotics modulate these pathways by altering gut microbiota composition.

The GBA is a bidirectional communication network connecting the gastrointestinal and CNS (Figure 1). It allows for exchanging information and coordinating various physiological processes (Hattori and Yamashiro, 2021). The gut microbiota, the trillions of microorganisms in the human gastrointestinal tract, plays a crucial role in the GBA (Tan, 2023). Healthy gut function has been linked to normal CNS function, as hormones, neurotransmitters, and immunological factors released from the gut can directly or indirectly send signals to the brain via the autonomic nervous system (ANS) (Clapp et al., 2017).

The existence of the GBA was first demonstrated in a landmark study that found impaired stress response in germ-free mice, suggesting that the gut microbiota is essential for developing a normal stress response (Clapp et al., 2017). This axis involves gastrointestinal motor and sensory components sending messages to the CNS, which can influence brain development and function (Hattori and Yamashiro, 2021).

The axis acts through neural, immunological, and endocrine pathways. In the neural pathway, the enteric nervous system (ENS) and the vagus nerve (VN) facilitate communication between the gut and the CNS (Zhu et al., 2017). Additionally, the microbiome can impact the production of neurochemicals such as serotonin, which has been linked to conditions like irritable bowel syndrome (Aszalós, 2008).

Gut bacteria are involved in the production of neurotransmitters like serotonin and GABA. Approximately 90–95% of the body's serotonin is produced in the gastrointestinal tract, primarily by enterochromaffin cells, and gut bacteria can influence this production (Yano et al., 2015). Certain bacteria, such as *Lactobacillus* and *Bifidobacterium*, produce GABA, which helps modulate anxiety and mood (Dinan and Cryan, 2017). Studies have shown that the gut microbiota can modulate the expression and sensitivity of neurotransmitter receptors, impacting emotional responses and stress management (Bruce-Keller et al., 2015). Moreover, the gut microbiota can influence the CNS by producing short-chain fatty acids (SCFAs), which affect neurotransmitter synthesis and receptor activity. SCFAs such as butyrate can enhance serotonin production and influence receptor expression (Silva et al., 2020).

Gut peptides such as ghrelin and leptin, which are involved in hunger regulation, also signal the brain. These peptides can influence cognitive functions and mood. Leptin can modulate depressive behavior by crossing the blood-brain barrier to interact with neurons in the hypothalamic arcuate nucleus, affecting the secretion of neuropeptides such as proopiomelanocortin (POMC) and neuropeptide Y (NPY), which play significant roles in mood regulation (Zarouna, 2015). Ghrelin administration in male rats improved mood and cognitive function (Jackson et al., 2019), increasing serotonin activity and receptor expression in key brain areas such as the amygdala and dorsal raphe (Hansson et al., 2014).

The immune system also plays a crucial role, with gut microbiota influencing inflammatory processes in the brain (Giau et al., 2018). Studies demonstrate alterations in the immune system and GBA function in association with various neurological and psychiatric disorders, including neurodevelopmental conditions, neurodegenerative diseases, and psychiatric illnesses (Long-Smith et al., 2020).

Disruptions in this axis, caused by poor diet, excessive antibiotic use, chronic stress, and gastrointestinal diseases, can exacerbate inflammation and depressive symptoms (Zhu et al., 2017; Long-Smith et al., 2020; Tan, 2023). A diet high in fat and sugar can negatively impact the gut microbiota, leading to increased inflammation and changes in brain function. A study by Bruce-Keller et al. found that mice fed a high-fat diet showed signs of inflammation and behavioral changes similar to depression. The research utilized a mouse model, where groups were fed either high-fat or control diets. The study's methodology involved microbiota transplantation from obese donors into germ-free mice and assessments of inflammation and behavioral changes using validated neurobehavioral tests. While not explicitly stated, the sample size likely followed standard experimental protocols for murine studies. The outcomes demonstrated that the high-fat diet induced significant inflammation and depressive-like behaviors, suggesting a gut-brain axis connection (Bruce-Keller et al., 2015).

Another research, by Korpela et al. demonstrated that children who received multiple courses of antibiotics had an increased risk of developing mental health issues. The authors examined the relationship between the intestinal microbiome and lifetime antibiotic use in Finnish preschool children. This research included a cohort of 142 children, with data collected longitudinally. Stool samples were analyzed using high-throughput sequencing to characterize the microbiome composition, while antibiotic usage was tracked through medical records. The findings revealed that children with multiple antibiotic courses exhibited an altered microbiome composition and an elevated risk of mental health issues, including anxiety and depression (Korpela et al., 2016).

The endocrine system and the gut microbiome are intricately linked, with the microbiome considered a full-fledged endocrine organ due to its various effects on the intestinal environment, which influences distant organs and pathways. The microbiota plays a crucial role in the reproductive endocrine system throughout a woman's lifetime by interacting with hormones such as estrogen, androgens, and insulin. Imbalances in gut microbiota composition can lead to several diseases and conditions, including pregnancy complications, adverse pregnancy outcomes, polycystic ovary syndrome (PCOS), endometriosis, and cancer (Qi et al., 2021). The relation between brain function and behavior and the endocrine system is actively being investigated. The emerging evidence underscores the importance of the GBA in regulating physiological and behavioral responses, particularly in stress (Foster et al., 2017; Long-Smith et al., 2020).

## Gut microbiota and depression

The GBA is crucial in maintaining bodily homeostasis and mental health. This axis is essential for the interaction between the gastrointestinal system and the brain, and imbalances in the gut microbiota, chronic inflammation, and stress can negatively affect brain function and mood (Du et al., 2020). Several studies have found that changes in the GBA modulate depressive symptoms. One of its related mechanisms is its role in synthesizing and metabolizing key neurotransmitters, including serotonin and dopamine, necessary for mood regulation and mental health (Huang and Wu, 2021).

Approximately 90% of the body's serotonin is synthesized in the gut by enterochromaffin cells, with gut bacteria influencing its production through various mechanisms. For instance, specific bacterial strains, such as *Lactobacillus* and *Bifidobacterium*, promote the synthesis of serotonin by modulating the availability of tryptophan, a precursor of serotonin (Yano et al., 2015; Valles-Colomer et al., 2019). Alterations in the GBA, such as those caused by dysbiosis, can disrupt serotonin levels, contributing to the development of depressive symptoms (Kelly et al., 2015).

Dopamine metabolism is also impacted by gut microbiota composition. Dopamine synthesis begins with the amino acid tyrosine, converted into L-DOPA by tyrosine hydroxylase. Certain gut bacteria, such as *Escherichia coli*, produce enzymes that influence dopamine metabolism. Dysbiosis can alter dopamine signaling by affecting the levels of its precursors and metabolites, leading to changes in reward-related behaviors and mood regulation (Cryan et al., 2019; Hamamah et al., 2022). These disruptions are increasingly recognized as potential contributors to the onset and progression of depression.

The GBA provides a bidirectional communication pathway through which gut microbiota regulate neurotransmitter production and vice versa. Influenced by gut bacteria, tryptophan metabolism plays a pivotal role in balancing the kynurenine and serotonin pathways. Chronic inflammation and gut dysbiosis have been shown to shift tryptophan metabolism toward the kynurenine pathway, increasing metabolites with neurotoxic properties, which is associated with neuroinflammation and depressive symptoms (Lukić et al., 2022; Mingoti et al., 2023).

Other changes in the gut microbiota have been associated with anxiety and depressive-like behaviors in humans (Tian et al., 2022; Zhu et al., 2023) and animal models (Guo et al., 2019; Cao et al., 2020). Evidence indicates that the microbiota of mice exposed to chronic stress increases neuroinflammation and contributes to these behaviors by altering bacterial composition and inflammatory cytokine activity in the brain (Li et al., 2019).

In humans, administration of the probiotic *Lactobacillus plantarum* JYLP-326 (Zhu et al., 2023) and *Bifidobacterium breve* CCFM1025 (Tian et al., 2022) showed significant antidepressant effects in patients with MDD. These effects were associated with modifications in the intestinal microbiota (Zhu et al., 2023) and in tryptophan metabolism, resulting in changes in the levels of serotonin and other relevant metabolites in serum, reflecting an improvement in the regulation of the GBA (Tian et al., 2022).

Emerging evidence suggests that alterations in the gut microbiome, known as dysbiosis, may contribute to depression (Rudzki and Szulc, 2018; Long-Smith et al., 2020). Dysbiosis can lead to increased intestinal permeability, which allows for the passage of inflammatory molecules and pathogens into the bloodstream, triggering an immune response that can adversely affect the brain (De Palma et al., 2015; Rudzki and Szulc, 2018). Chronic stress, a significant risk factor for depression, has also been shown to disrupt the gut microbiome, further exacerbating the problem (Tan, 2023).

While chronic stress may contribute to exacerbating inflammation associated with depression, the composition of the gut microbiota also plays a crucial role in this process, influencing both inflammatory factors and symptom severity. Studies show that stress, central and peripheral inflammation, and alterations in the gut microbiota are important risk factors for MDD (Cruz-Pereira et al., 2020; Pearson-Leary et al., 2020).

The gut microbiota plays a crucial role in regulating intestinal permeability, which may contribute to the chronic low-grade inflammation observed in disorders such as depression. Stress and depression can alter the composition of gut bacteria, which may influence eating behavior and mood. These changes in the intestinal microbiota may, in turn, increase the risk of developing depression, highlighting the connection between intestinal health and psychiatric disorders (Kelly et al., 2015; Madison and Kiecolt-Glaser, 2019).

Inflammatory depression, a treatment-resistant subtype, is associated with an altered composition of the gut microbiota, including higher levels of Bacteroides and lower levels of *Clostridium*, as well as an increase in SCFA-producing species with abnormal butanoate metabolism. Supplementation with *Clostridium butyricum* has been shown to normalize the gut microbiota, reduce inflammatory factors, and exhibit antidepressant effects in a murine model of inflammatory depression, suggesting that inflammatory processes derived from the gut microbiota may be involved in the neuroinflammation associated with inflammatory depression (Liu P. et al., 2024).

In animals, chronic stress disrupts the balance of the intestinal microbiota. It stimulates inflammatory mechanisms in the brain, leading to anxiety disorders and MDD. At the same time, *Bifidobacterium adolescentis* demonstrates anxiolytic and antidepressant effects by reducing the inflammatory cytokines IL-1β and TNF-α, in addition to decreasing the expression of p-nuclear factor-kappa B (NF-κB) p65 and Ionized calcium-binding adaptor molecule 1 (Iba1), and by rebalancing the intestinal microbiota (Guo et al., 2019).

Another substance with potential antidepressant effects by regulating the GBA, altering the intestinal microbiota, and reducing LPS and inflammatory cytokines is Kai-Xin-San (KXS). In mice with chronic stress-induced depression, KXS increased the expression of tight junction proteins in the gut barrier and blood-brain barrier and decreased stress-related hormones in the CNS. Antibiotic administration attenuated the antidepressant effects of KXS, indicating that its benefits are linked to the modulation of inflammation and regulation of the gut microbiota (Cao et al., 2020).

### The HPA axis and the GBA

**Figure 2:**
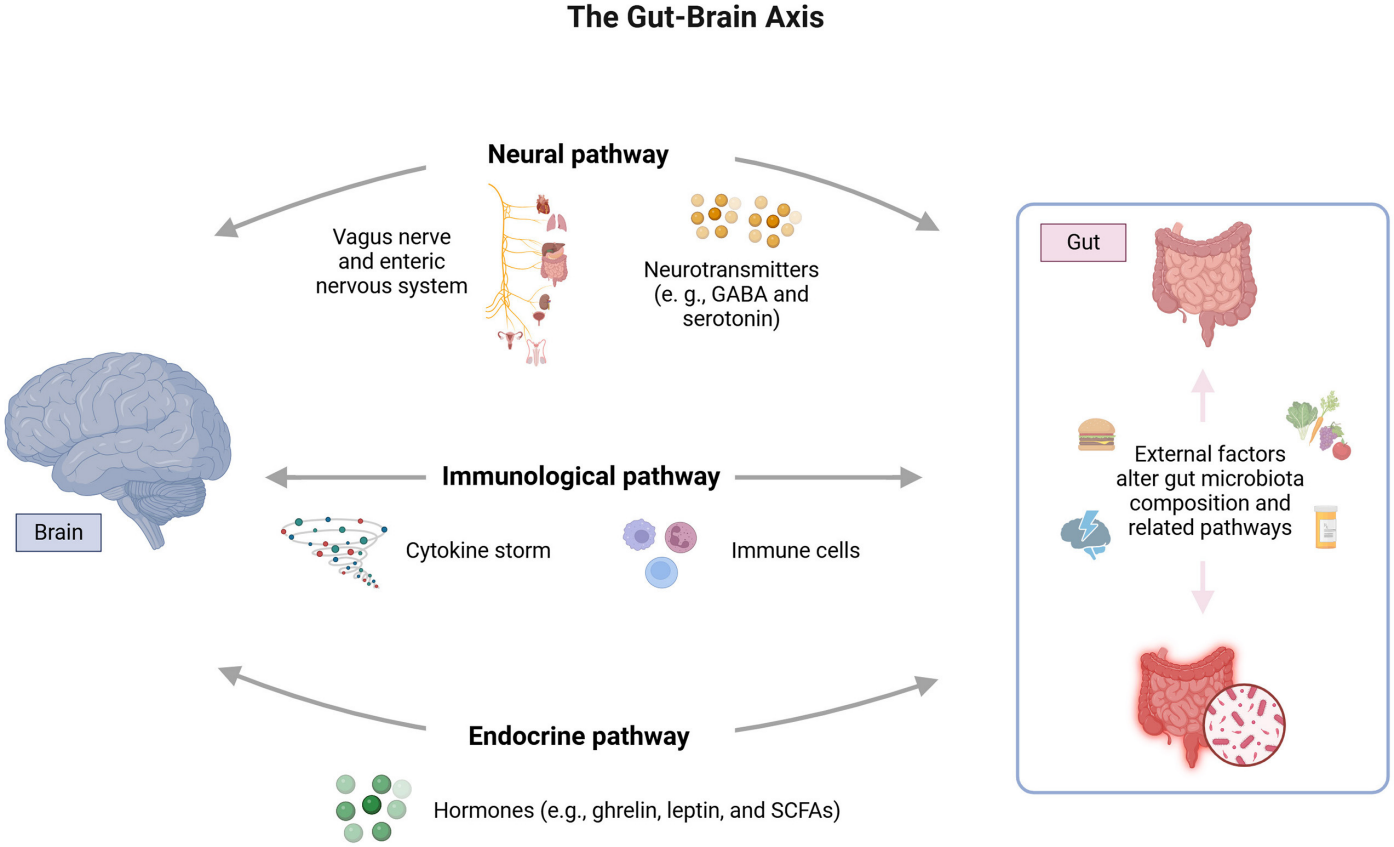
Bidirectional interaction between the HPA axis and the gut-brain axis (GBA). The HPA axis responds to stress by releasing glucocorticoids, such as cortisol, which modulate the intestinal barrier, IgA production, and gut microbiota. The intestinal barrier, composed of epithelial cells and tight junctions, is crucial to preventing the translocation of pathogens and toxins. Microbiota can influence the HPA axis through immune molecules, hormones and neurotransmitters.

The HPA axis is activated in response to stress, releasing hormones such as cortisol, which help the body cope with adverse situations. On the other hand, the GBA regulates the bidirectional communication between the brain and the gut, involving the CNS, the enteric nervous system, and the gut microbiota (Figure 2). Studies show that stress can alter the intestinal microbial composition, while changes in the microbiota can, in turn, influence HPA axis activity (Foster et al., 2017).

The HPA axis, by releasing glucocorticoids in response to internal and external stimuli, directly influences the production of immunoglobulin A (IgA) in the gastrointestinal tract. This immunoglobulin is essential for protecting the intestinal mucosa, and neutralizing pathogens and toxins while preserving microbiota homeostasis. Studies highlight that alterations in HPA axis activity, as seen in specific stress situations, can reduce IgA levels, compromising the body's ability to preventively respond to external threats and destabilizing microbial balance (Rea et al., 2020).

Furthermore, the intestinal barrier, composed of a layer of epithelial cells interconnected by tight junctions, is a critical structure for separating the intestinal lumen from the circulatory system (Rea et al., 2020). The glucocorticoids released by the HPA axis can modulate the expression of tight junction proteins, such as occludin and claudin (Rusch et al., 2023). In conditions of HPA axis hyperactivity, observed in inflammatory or infectious contexts, this regulation is disrupted, resulting in increased intestinal permeability. This facilitates the translocation of microorganisms, toxins, and pro-inflammatory molecules into the bloodstream, activating systemic inflammatory responses (Rusch et al., 2023).

The activation of the HPA axis during infections alters the composition of the gut microbiota and the production of antimicrobial metabolites (Rusch et al., 2023). This interaction is essential for modulating the host immune response and maintaining microbiota functionality during infectious episodes. For example, during bacterial infections, the HPA axis response can stimulate the production of specific metabolites that contain invasive pathogens while preserving beneficial microbiota species (Thaiss et al., 2016).

Diet and lifestyle modulate the GBA and HPA axis. A balanced diet rich in fiber and low in processed foods, combined with healthy lifestyle practices, can promote a healthy gut microbiota and stable HPA axis, potentially reducing the risk of chronic diseases and stress-related disorders (Rusch et al., 2023).

Diet, particularly macronutrients like fibers, proteins, and fats, play a major role in gut microbiota shaping. High-fiber diets promote beneficial bacteria and the production of short-chain fatty acids, which are important for gut health and reducing inflammation (Conlon and Bird, 2014; De Angelis et al., 2019; Hills et al., 2022). Conversely, Western diets high in fats and proteins and low in fibers can lead to dysbiosis, promoting inflammation and metabolic dysfunction (Moschen et al., 2012; Rinninella et al., 2019; Barber et al., 2021). The gut microbiota communicates with the brain through the GBA, influencing the HPA axis (Cryan et al., 2019; Simkin, 2019; Barber et al., 2021). For instance, diets rich in plant polysaccharides can enhance microbiota diversity and functionality, potentially stabilizing the HPA axis and reducing stress-related symptoms (Cryan et al., 2019; Hills et al., 2022).

Lifestyle elements such as stress, sleep, and exercise also affect gut microbiota. Stress, particularly in athletes, can alter gut microbiota composition, impact immune function, and potentially lead to gastrointestinal issues (Clark et al., 2016; Redondo-Useros et al., 2020). Modern lifestyle habits, including processed food consumption and lack of sleep, contribute to gut dysbiosis and related health issues (Simkin, 2019; Redondo-Useros et al., 2020).

Stress profoundly impacts the bidirectional communication between the HPA axis and the gut microbiota through the GBA. Activation of the HPA axis during stress increases glucocorticoid levels, such as cortisol, which can disrupt gut microbiota composition and compromise intestinal barrier integrity. This allows microbial components like LPS to enter circulation, promoting systemic inflammation and worsening gut dysbiosis (Sudo et al., 2004).

Conversely, dysbiotic gut microbiota can influence the HPA axis by reducing the production of metabolites such as SCFAs, which are essential for neuroimmune signaling and stress regulation (Moloney et al., 2014). Dysbiosis also affects neurotransmitter synthesis, such as GABA, intensifying the stress response (Cryan and Dinan, 2012). Strategies such as probiotics and dietary changes have shown the potential to modulate this communication. Probiotic strains like *Lactobacillus* and *Bifidobacterium* can help restore microbiota balance, reduce cortisol levels, and support HPA axis stability (Messaoudi et al., 2011).

### Interaction between the HPA axis, gut-brain and biological mechanisms involved in depression

**Figure 3:**
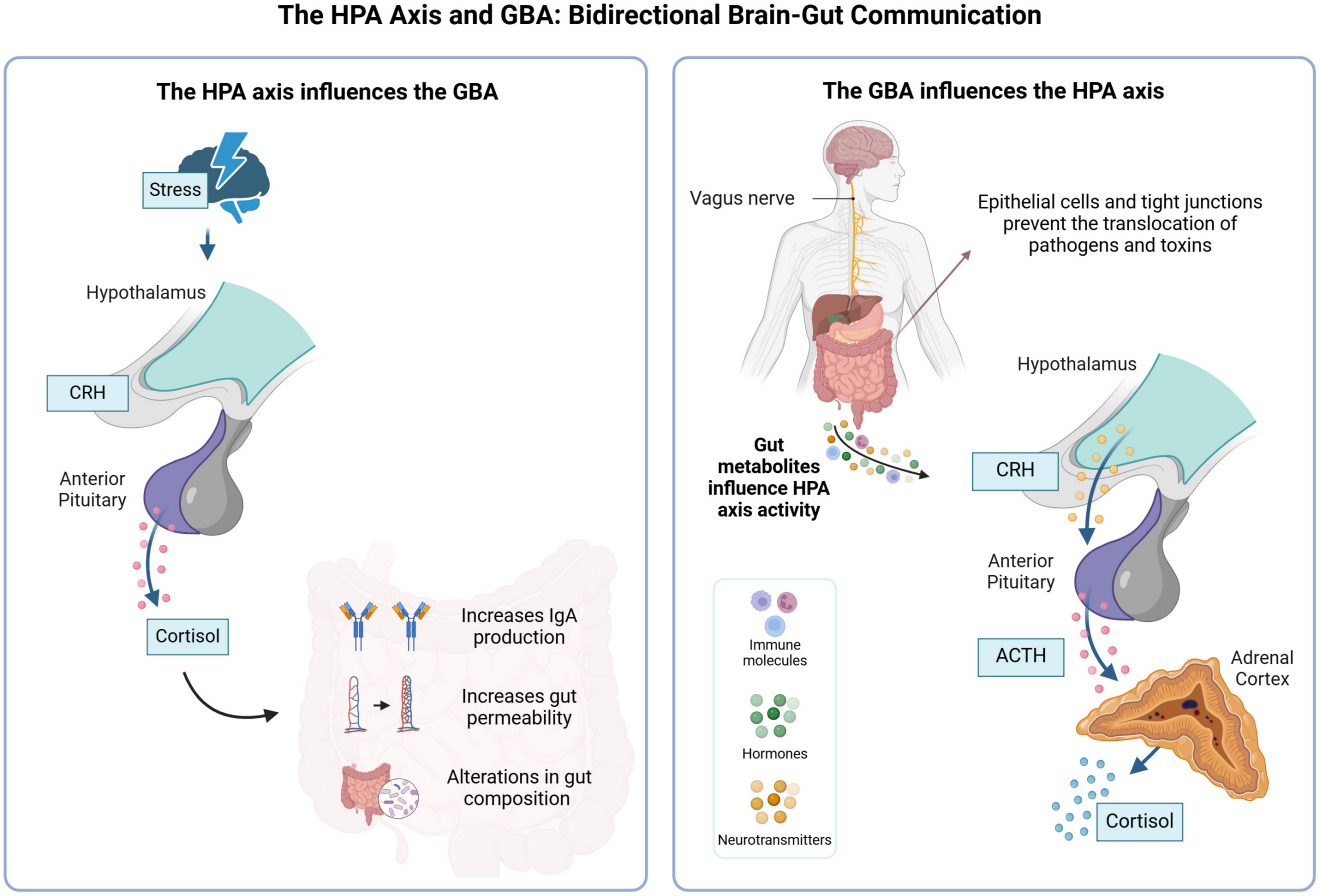
Communication between the gut-brain axis, HPA dysregulation, and depression. Gut dysbiosis contributes to changes in the release of neurotransmitters, gut peptides, cytokines, and microbial antigens, which influence the hypothalamic-pituitary-adrenal (HPA) axis. The hypothalamus releases corticotropin-releasing hormone (CRH), stimulating the anterior pituitary to produce adrenocorticotropic hormone (ACTH), which stimulates the release of cortisol by the adrenal cortex. Dysbiosis can result in chronic alterations in the HPA axis, impairing axis regulation and chronic excess cortisol in the circulation. Excess cortisol can culminate in neuroinflammation and brain changes, such as reduced neuronal plasticity, decreased brain-derived neurotrophic factor (BDNF) levels, and atrophy. All these changes are mostly present in the hippocampus and prefrontal cortex and contribute to cognitive deficits, depressive symptoms, and impaired adaptation to stress. Chronic stress and HPA dysregulation further exacerbate gut dysbiosis, creating a pathological feedback loop. The blue arrows indicate the communication pathways between the different components of the gut-brain axis and the HPA axis. The granules represent indirect or molecule-mediated influences, such as neurotransmitters, gut peptides, and cytokines. The light blue circular area at the bottom represents the pathological feedback loop between gut dysbiosis and HPA axis dysregulation.

The bidirectional communication of the GBA, a pivotal regulator of the HPA axis, is deeply involved in the development of MDD (Figure 3; Makris et al., 2021). The gut microbiota, through its influence on the HPA axis or alteration of its composition, potentially via neurotransmitters, gut peptides, and immune system activation, significantly affects this process (Młynarska et al., 2022). Furthermore, the impact of mediators from the gut microbiota that cross the blood-brain barrier on HPA axis activity and gut-brain communication, particularly in severe mental disorders, is a significant area of study (Misiak et al., 2020).

The increased activity of the HPA axis leads to greater secretion of cortisol, which contributes to the elevation of inflammatory biomarkers. One explanation for the cause of these disorders is that, with chronic stress and MDD, there is a dysfunction of glucocorticoid receptors that impairs the negative feedback of the HPA axis. This affects the glucocorticoid receptors in the hypothalamus and pituitary gland (Faria and Longui, 2006), potentially resulting in glucocorticoid resistance (Neufeld et al., 2011; Nikkheslat et al., 2020).

Glucocorticoids are steroid hormones secreted by the adrenal cortex and are also related to the circadian cycle (Nikkheslat et al., 2020). Hormonal secretion triggers different responses in the body. In memory consolidation and learning processes, glucocorticoids, in conjunction with their receptors, modulate brain areas involved in these processes, including the hippocampus, amygdala, and prefrontal cortex (Groeneweg et al., 2012).

Glucocorticoids are involved in fundamental metabolic processes in the human body and are regulated through the HPA axis (Nicolaides et al., 2010). Glucocorticoids also control and maintain basal homeostasis in stressful situations (Galon et al., 2002; Faria and Longui, 2006). Approximately 20% of the genes expressed in leukocytes are positively or negatively regulated by glucocorticoids in humans (Galon et al., 2002).

When there is intense or chronic exposure to stress, brain homeostasis, particularly in those brain regions closely related to glucocorticoids, can be affected, resulting in deficits in neuronal neuroplasticity that disadvantage the affected individual in dealing with new stressful situations (McEwen and Gianaros, 2010). Moreover, the chronic elevation of glucocorticoids desensitizes the activation of their receptors, rendering them resistant in patients with a recurrent and immature history of stress associated with MDD (Fernández-Guasti et al., 2012).

Sustained activation of the HPA axis leads to excessive release of glucocorticoids, which under normal conditions have an anti-inflammatory effect. However, the chronic inflammatory state induces glucocorticoid resistance, resulting in the perpetuation of the inflammatory response and regulatory dysfunction of the HPA axis (Knezevic et al., 2023).

Chronic inflammatory stimuli induce epigenetic modifications, such as methylation of inflammatory genes and alterations in histone acetylation. These changes stabilize pro-inflammatory states and sensitize the immune and nervous systems to new stressors, increasing the risk of depressive episodes (Stankiewicz et al., 2013).

Stress can lead to the chronic sensitization of an individual to the secretion of hormones such as CRH, which in turn influences the increase of pituitary ACTH. The release of these hormones triggers the biosynthesis and release of glucocorticoids in the adrenal cortex (Fernández-Guasti et al., 2012).

Boyle et al. (2005) reinforce this hypothesis by finding that rodents with glucocorticoid receptor dysfunction exhibited increased HPA activity and behaviors related to depressive and anxious states. Additionally, the interaction between the HPA axis and the GBA is crucial in modulating inflammation and impacting mental health. Hyperactivity of the HPA axis in response to chronic stress can result in resistance to the effects of cortisol. Combined with an imbalanced gut microbiota that promotes systemic and local inflammation, this can amplify neuroinflammation in the brain, exacerbating depressive symptoms (Black and Garbutt, 2002; Dantzer et al., 2008; Mayer et al., 2015).

Similarly, gut dysbiosis can influence HPA axis activity and, consequently, the production of inflammatory mediators, exacerbating depressive symptoms. The gut microbiota can activate the HPA axis through mediators such as microbial antigens, cytokines, prostaglandins, neurotransmitters, gut peptides, and immune system activation, impacting HPA axis activity and intestinal permeability in severe mental disorders. Biological mechanisms, such as altered intestinal permeability and systemic inflammatory response, have been associated with greater susceptibility to emotional disorders (Misiak et al., 2020; Młynarska et al., 2022).

Increased cortisol levels due to HPA axis hyperactivity can negatively affect the production and availability of neurotransmitters such as serotonin and glutamate. Simultaneously, changes in the intestinal microbiota can alter the synthesis and metabolism of neurotransmitters (Holsboer, 2000; Foster and McVey Neufeld, 2013), such as by reducing the availability of tryptophan, an essential precursor for the production of serotonin (Stephens and Wand, 2012), in addition to influencing the function of glutamate receptors and influencing the release and uptake of this neurotransmitter (McEwen, 2007), contributing to a depressive state.

Therefore, systemic inflammation alters the metabolism of tryptophan by activating the enzyme indoleamine 2,3-dioxygenase (IDO). This process diverts tryptophan to the kynurenine pathway, which is responsible for the synthesis of serotonin. Neurotoxic metabolites, such as quinolinic acid, are offered and reduced for excitotoxicity and oxidative stress, worsening depressive symptoms (Pariante and Lightman, 2008).

Chronic stress and hyperactivity of the HPA axis can induce atrophy of brain regions crucial for neuronal plasticity, such as the hippocampus. Studies show that excess cortisol associated with prolonged stress causes a reduction in neurogenesis and dendrite density in the hippocampus, contributing to cognitive deficits and depressive symptoms (McEwen, 2007). Furthermore, alterations in the intestinal microbiota can amplify this neuronal dysfunction. Intestinal dysbiosis can increase systemic and neuronal inflammation, affecting the production and availability of neurotransmitters, such as serotonin and glutamate, and exacerbating the neuronal dysfunction associated with depression (Cryan and Dinan, 2012).

BDNF is essential for neurons' survival, growth, and plasticity, with higher levels associated with better mental health and reduced vulnerability to depression. Decreased BDNF levels, often observed in individuals with MDD, are related to atrophy of brain areas such as the hippocampus (Duman and Monteggia, 2006). The HPA axis regulates the stress response and, when overactive, elevates cortisol levels, which can cause atrophy of brain regions crucial for neuronal plasticity and reduce the availability of BDNF, exacerbating depression (De Kloet et al., 2005). The gut microbiota, which communicates with the brain through the GBA, can, when altered (dysbiosis), increase systemic and neuronal inflammation, affect neurotransmitter production, and negatively impact BDNF function. Gut dysbiosis can also contribute to HPA axis dysfunction, increasing vulnerability to depression (Dinan and Cryan, 2017).

Specific bacteria and their metabolites significantly influence the HPA axis and neurotransmitter levels through multiple mechanisms. Microbial components, such as LPS, can activate the HPA axis by triggering inflammatory responses mediated by cytokines like IL-6 and TNF-α, which cross the blood-brain barrier and stimulate the release of stress hormones such as cortisol or corticosterone (Zimomra et al., 2011; Misiak et al., 2020). Stress-induced bacterial translocation, often involving pathogens like *Salmonella*, further exacerbates HPA axis activation by increasing corticosterone levels (Ando et al., 2000). Prostaglandins produced during bacterial challenges facilitate the initial rise in corticosterone, while cytokines maintain this response over time (Zimomra et al., 2011).

In addition to their effects on the HPA axis, specific bacteria influence neurotransmitter levels through direct and indirect pathways. Certain strains, including *Lactobacillus* and *Bifidobacterium*, produce neurotransmitters such as GABA and serotonin, which modulate neural signaling and mood (Misiak et al., 2020; Horvath et al., 2021). LPS from bacterial origin can also alter the metabolism of serotonin and catecholamines in the CNS, impacting behavior and emotional responses (Linthorst et al., 1995; Ando et al., 2000). Furthermore, bacteria like *Bacteroides* modulate intestinal neurotransmitter levels, communicating with the brain via the vagus nerve and shaping cognitive and emotional processes (Horvath et al., 2021).

HPA axis dysfunction, combined with alterations in the gut microbiota, can create an environment conducive to dysregulated stress responses. Chronic stress can amplify the effects of an imbalanced microbiota, potentiating inflammation and negatively affecting neurotransmitter function. Furthermore, this combination can lead to atrophy of brain tissues, such as the hippocampus, further impairing neuronal plasticity. The interaction between prolonged stress, gut dysbiosis, and brain atrophy can decrease levels of BDNF, a crucial factor for neuron survival and growth, exacerbating the inflammatory and neurotransmitter response and significantly increasing vulnerability to MDD.

To apply the findings to clinical practice, it is recommended that clinicians consider both the HPA and gut-brain axes as essential components in the treatment of depression. Interventions focused on reducing chronic stress and modulating inflammation, such as mindfulness-based stress reduction, anti-inflammatory treatments, and nutritional strategies (including the use of probiotics, prebiotics, and fiber-rich diets), should be integrated into therapeutic approaches (Rudzki and Szulc, 2018; Tian et al., 2022; Zhu et al., 2023). These strategies can help regulate cortisol levels, support gut microbiota balance, and enhance neurotransmitter production, particularly serotonin (Cao et al., 2020; Li et al., 2019). Additionally, exploring emerging therapies like fecal microbiota transplantation (FMT) may provide valuable options for patients with treatment-resistant depression (Cao et al., 2020; Long-Smith et al., 2020). Addressing these interconnected pathways can more effectively target the biological mechanisms of depression and improve patient outcomes.

## Considerations and future directions

The interaction between the HPA axis and the GBA plays a significant role in the biological pathways that contribute to depression. Research indicates that imbalances in the gut microbiota, chronic inflammation, and stress can profoundly affect brain function and mood regulation. Studies show that alterations in gut microbiota composition are linked to anxiety and depression-like behaviors in both humans and animal models. Dysbiosis, marked by changes in gut microbiota, has been connected to increased intestinal permeability, allowing inflammatory molecules to enter the bloodstream and trigger immune responses that harm the brain. Chronic stress worsens these effects by disrupting gut microbiota, further contributing to the onset and persistence of depression.

Gut microbiota's role in regulating intestinal permeability and inflammation helps explain its impact on mental health. For example, changes in microbiota diversity and imbalances in key bacterial species have been identified in inflammatory depression, which is often resistant to conventional treatments. Emerging therapies targeting the gut microbiota, including probiotics and microbiota-modulating compounds, have shown promise in alleviating depressive symptoms by enhancing gut barrier function, reducing inflammation, and regulating neurotransmitter synthesis. Specific probiotic strains have been shown to alleviate depressive symptoms by influencing gut microbiota and tryptophan metabolism, which directly impacts serotonin levels.

Further exploring the connection between the HPA axis and gut microbiota could enhance understanding of stress regulation and its influence on mental health. Studying individual differences in microbiota composition and stress responses could pave the way for developing personalized treatment approaches. Emerging technologies such as FMT and precision medicine offer promising avenues for addressing depression by effectively targeting the gut microbiota and HPA axis. Additionally, developing novel probiotics, tailored to modulate the gut microbiota more precisely, may lead to more targeted interventions for depression.

Collaboration among neuroscience, psychiatry, and microbiology is essential for advancing knowledge. Continued research could lead to more effective and comprehensive treatments, ultimately improving outcomes for individuals suffering from depression.
